# Prism adaptation does not alter object-based attention in healthy participants

**DOI:** 10.12688/f1000research.2-232.v1

**Published:** 2013-11-04

**Authors:** Janet H. Bultitude, Alexandra List, Anne M. Aimola Davies

**Affiliations:** 1 Oxford Centre for Functional Magnetic Resonance Imaging of the Brain (FMRIB), Nuffield Department of Clinical Neurosciences, University of Oxford, Oxford, OX3 9DU, UK; 2 Department of Psychology and Neuroscience Program, Hamilton College, Clinton, NY 13323, USA; 3 Research School of Psychology, The Australian National University, Canberra, 0200, Australia; 4 Department of Experimental Psychology, University of Oxford, Oxford, OX1 3UD, UK

## Abstract

Hemispatial neglect (‘neglect’) is a disabling condition that can follow damage to the right side of the brain, in which patients show difficulty in responding to or orienting towards objects and events that occur on the left side of space. Symptoms of neglect can manifest in both space- and object-based frames of reference. Although patients can show a combination of these two forms of neglect, they are considered separable and have distinct neurological bases. In recent years considerable evidence has emerged to demonstrate that spatial symptoms of neglect can be reduced by an intervention called prism adaptation. Patients point towards objects viewed through prismatic lenses that shift the visual image to the right. Approximately five minutes of repeated pointing results in a leftward recalibration of pointing and improved performance on standard clinical tests for neglect. The understanding of prism adaptation has also been advanced through studies of healthy participants, in whom adaptation to leftward prismatic shifts results in temporary neglect-like performance. Here we examined the effect of prism adaptation on the performance of healthy participants who completed a computerised test of space- and object-based attention. Participants underwent adaptation to leftward- or rightward-shifting prisms, or performed neutral pointing according to a between-groups design. Significant pointing after-effects were found for both prism groups, indicating successful adaptation. In addition, the results of the computerised test revealed larger reaction-time costs associated with shifts of attention between two objects compared to shifts of attention within the same object, replicating previous work. However there were no differences in the performance of the three groups, indicating that prism adaptation did not influence space- or object-based attention for this task. When combined with existing literature, the results are consistent with the proposal that prism adaptation may only perturb cognitive functions for which normal baseline performance is already biased.

## Introduction

Hemispatial neglect (‘neglect’) is a disabling condition that can follow brain injury. Patients with neglect show difficulty in responding to or orienting towards objects and events that occur on the contralesional side of space ^1^. Symptoms of neglect can be reduced following a brief period of a sensory-motor training technique called prism adaptation ^2^. Patients who spend a few minutes pointing to visual targets while wearing prismatic glasses that shifted the visual image to the right demonstrate a leftward shift, or ‘adaptation’, in their pointing as well as improved performance on standard clinical tests such as target cancellation and line bisection. The benefits of prism adaptation have been observed in multiple sensory modalities: vision ^3^, tactile detection ^4^, haptic exploration ^5^, pressure sensitivity and finger position sense ^6^, and auditory processing ^7^. Furthermore, prism adaptation results in improvements in tasks that have direct relevance for recovery of independence such as reading ^8, 9^, postural control ^10^, and wheelchair navigation ^11, 12^. The broad generalisation of prism adaptation treatment has generated considerable interest in understanding the mechanisms by which the technique reduces symptoms.

In healthy participants, adaptation to leftward-shifting prisms (producing a rightward reaching bias) temporarily induces neglect-like performance on tests of lateralised spatial attention such as a non-manual version of the line bisection test called the landmark test ^13– 17^. Although the changes shown by healthy participants are generally smaller in magnitude than those shown by neglect patients, they too have been observed in multiple sensory modalities ^14, 16– 19^. Interestingly, Michel and colleagues ^13^ showed that the magnitude of midpoint shift increased with more leftward line placement and longer line length, replicating the so-called ‘position’ and ‘length’ effects that have been described in neglect patients. Therefore, although adaptation to rightward-shifting prisms can reduce symptoms in neglect patients by shifting the response bias towards the left side of space, adaptation to leftward-shifting prisms can induce a similar but opposite change in healthy participant, inducing a small rightward response bias. The similarity in the effects of prism adaptation on the performance of neglect patients and healthy controls makes it possible to gain insights into the potential therapeutic effects of the technique through experiments on healthy volunteers.

The primary motivation of the present study was to investigate whether prism adaptation can alter object-based attention: that is, the extent to which object boundaries influence the allocation and redirection of selective attention. Considerable research provides support for the existence of object-based attention mechanisms that are distinct from a purely spatially-based allocation of attention in which object boundaries are irrelevant ^20– 22^. A particularly compelling demonstration of object-based attention was provided by Egly, Driver and Rafal ^23^. In their task (the ‘Egly task’), two rectangles were arranged horizontally or vertically on either side of a central fixation cross. Targets appeared at one end of one rectangle. The targets were preceded by a cue in either the same location (validly-cued) or a different location (invalidly-cued) to the target. Critically, for invalidly-cued trials the cue location was either at the same end of the adjacent rectangle as the target (requiring a between-object shift of attention), or in the opposite end of the same rectangle as the target (requiring a within-object shift of attention). By setting the length of the rectangles to be equidistant to the distance separating the rectangles, the authors ensured that any difference in reaction times (RT) for the two invalidly-cued conditions could only be attributed to object-based attention mechanisms (because the spatial separation of the cue and target locations was the same across conditions). Larger RT costs were found for the between-object condition than for the within-object condition, providing evidence for object-based attention.

In neglect patients, symptoms can manifest in both space- and object-based frames of reference. For example, a neglect patient may fail to copy all objects on the left side of a page (space-based neglect), or may fail to copy the left half of every object in the scene regardless of where they appear on the page (object-based neglect). Although patients often demonstrate a combination of space- and object-based neglect, the two are considered to be separable and have partially distinct neurological bases ^24, 25^. The broad generalisation of benefits induced by prism adaptation suggests that the therapy can influence core aspects of neglect symptoms. However, although several studies suggest that prism adaptation can alter space-based orienting in neglect patients ^26– 29^ and healthy participants ^14– 16^, there is relatively little evidence that prism adaptation would have equal efficacy in treating patients whose deficits are primarily object-based.

Some insights into whether prism adaptation can influence object-based attention were provided by Schindler and colleagues ^30^. Healthy participants, right-hemisphere lesioned patients *without* neglect, and right-hemisphere patients *with* neglect completed the Egly task before and after adaptation to rightward-shifting prisms. Their task had a similar format to the original Egly task, in that there were two rectangles presented either vertically or horizontally, and these were positioned one on each side of a central fixation cross. Compared to the two control groups, the neglect patients were significantly worse at shifting their attention from one object in the ipsilesional field to another in the contralesional visual field, and this deficit was abolished by prism adaptation. This finding suggests that prism adaptation can influence object-based attention. However there are still some outstanding questions regarding both object-based attention and how it is influenced by prism adaptation. First, for healthy participants adaptation to leftward-shifting prisms can alter cognitive performance, however adaptation to rightward-shifting prisms generally has no such effect (although see Berberovic and Mattingley ^19^ for an exception). Because Schindler and colleagues only tested adaptation to rightward-shifting prisms, it is not yet known whether prism adaptation can induce neglect-like changes in object-based attention in healthy participants. Second, the object-based deficit reported by Schindler and colleagues is potentially confounded by the requirement to shift attention between the two visual fields. Neglect patients were slower at shifting their attention between-objects only when it required a shift of attention from the ipsilesional to contralesional field: the RTs of neglect patients were normal for between-object shifts vertically *within* a visual field, and for horizontal within-object shifts from the ipsilesional to contralesional field. It is therefore unclear whether the pattern shown by their neglect patients at baseline can be attributed to a general deficit in shifting attention horizontally between two objects, or to a more specific deficit in shifting attention between two objects *that are presented in different visual hemifields*. By extension, it is unclear whether the improvements observed following prism adaptation were driven by a change in object-based attention, or an improvement in inter-hemisphere signalling.

In the present study we examine the effect of prism adaptation on space- and object-based attention in healthy participants using an adapted version of the Egly paradigm ^31^ in which all shifts of attention were restricted within one of the two visual fields (see Figure 2). Four rectangles (two in each visual field) were presented either horizontally or vertically in each trial. Cues and targets for each trial appeared in the same visual field according to three conditions (validly-cued, within-object shift and between-object shift). In a between-group design, participants completed blocks of the Egly task after adaptation to leftward- or rightward-shifting prisms, or neutral pointing.

Using this four-rectangle version of the Egly paradigm we examined the effect of prism adaptation on three aspects of attention that are known to be altered in hemispatial neglect. First, we examined the effect of prism adaptation on object-based attention by comparing reaction times for validly-cued trials, within-object trials and between-object trials in each visual field across the three participant groups.

Second, we examined the effect of prism adaptation on the tonic distribution of attention across the visual field. Kinsbourne ^32^ argued that the distribution of visuo-spatial attention across space is determined by two opposing gradients controlled by the contralateral cerebral hemispheres. One possible way that prism adaptation influences attention is by altering the balance between these opposing gradients: i.e., by rebalancing the hemispheric competition in patients and unbalancing the hemispheric competition in healthy controls. If this is the case, then adaptation to leftward-shifting prisms would induce a neglect-like gradient of attention in healthy participants, with lowest attention (highest RTs) to the left-most part of the visual field, and highest attention (lowest RTs) to the right-most part of the visual field.

Finally, we examined the effect of prism adaptation on horizontal shifts of attention. Patients with neglect are impaired at shifting their attention in a contralesional direction (the ‘disengage deficit’ ^33^). There is some evidence that prism adaptation can reduce the disengage deficit in neglect patients ^28, 34^ and can alter reaction times for horizontal shifts of attention in healthy participants ^35^, although there are some inconsistencies in the effects that have been observed for different cue types across these studies. We predicted that adaptation to leftward-shifting prisms would result in a neglect-like deficit in shifting attention leftward. This neglect-like deficit may be restricted to between-object shifts of attention, in keeping with the findings of Schindler and colleagues ^30^.

## Methods

### Participants

Sixty healthy undergraduates (13 males) participated in the experiment in exchange for course credits (mean age=19.6 years, *SEM*=0.3). Each participant had normal or corrected-to-normal vision and was right-handed according to the Edinburgh Handedness Inventory (mean=-0.84, SEM=0.02, where -1 denotes exclusive right-handedness ^36^). Informed consent was obtained in accordance with guidelines approved by the Bangor University ethics committee and the 2008 Declaration of Helsinki.

### Stimuli and procedure

**Figure 1.**
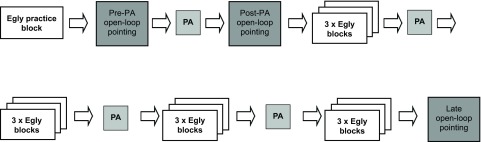
General procedure. PA = prism adaptation.

The general procedure is outlined in Figure 1. Throughout the experiment the participant was seated in a standard computer chair that could be wheeled and rotated by the experimenter between the computer and a custom-built prism adaptation box. The participant completed the Egly task in four sets of three blocks (twelve blocks in total), with prism adaptation preceding each set. Multiple sets of prism adaptation were used to ensure that the participant was fully adapted throughout all of the Egly blocks. Similar ‘top-up’ adaptation sets have been used previously ^30, 37^. The participant completed one practice Egly block before the first sham adaptation session during which the experimenter gave verbal feedback on the participant’s performance. To confirm visuo-motor adaptation, open-loop pointing tests were performed immediately before and after the first session of prism adaptation, and at the end of the experiment (the ‘pre’, ‘post’ and ‘late’ open-loop pointing sessions, respectively). Prism direction was manipulated between groups, with each participant randomly allocated to undergo adaptation to neutral, leftward-shifting or rightward-shifting lenses.

### Prism adaptation

For prism adaptation, the participant faced a 90 cm wide × 35 cm high × 70 cm deep prism adaptation box. The box was open at two opposite ends at which the participant and experimenter were positioned, as well as at the top, allowing the participant vision of the inside of the box. Three 1.5 cm diameter targets were placed on the base of the box at arm’s length from the participant and at angles of -10°, 0° and +10° from their body midline (negative numbers indicate a leftward displacement). The participant was fitted with welding goggles containing neutral lenses, or 25-diopter (~17°) Fresnel lenses that induced a visual shift to the left or right. While resting their chin on the edge of the box, the participant reached out to touch the targets in a pre-determined sequence (left-middle-right-middle) that was repeated for 90 pointing movements, returning their hand to rest in front of their torso between each movement. The participants could see only the distal half of each reaching movement. Pointing was performed in time with a metronome set to 1 Hz to encourage a constant, ballistic pointing speed. After completing the 90 pointing movements, the participant closed their eyes and the goggles were removed. To minimise de-adaptation, the participant was asked to keep their eyes closed between the different parts of the experiment, with the exception that after each set of three Egly blocks a more extended break was offered in which the participant could keep their eyes open before undergoing another set of prism adaptation.

### Pre-adaptation, post-adaptation and late open-loop pointing

A lid was placed on the box with lines drawn on the upper surface radiating at angles of -10°, 0° and +10° from the participant’s body midline. These served as target lines for the open-loop pointing task. The participant rested their chin on the top of the box and pointed with their right arm under each of the target lines four times in pseudorandom order as directed by the experimenter, returning their hand to rest in front of their torso between each pointing movement. The participant was instructed to point with their elbow straight and their index finger extended. Pointing error was measured by the experimenter to the nearest 0.5 degrees with the aid of markings drawn on the underside of the lid.

### Egly task

Stimuli were generated by Eprime software on a Dell PC running Windows XP. They appeared on a 17-inch monitor running at 85 Hz, positioned 60 cm from the participant’s eyes. Head movements were prevented by a chin rest that also prevented vision of the responding (right) hand.

**Figure 2.**
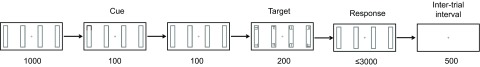
Trial timecourse. The figure shows the timing and duration of each event (ms). A vertical arrangement of rectangles (as shown) was used in half of the trials, and a horizontal arrangement was used in the remaining half of the trials. A within-object condition is shown.

**Figure 3.**
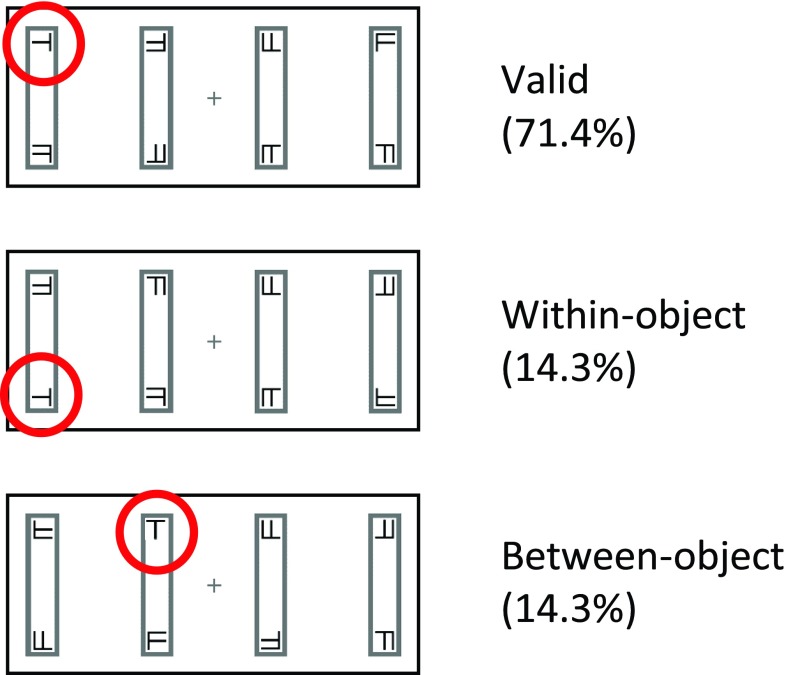
Possible target locations. The three possible target locations are shown relative to the cue location given in Figure 2. For the three possible trial types the target ‘T’ is circled in red for illustrative purposes (no red circle was displayed in the experiment).

Figure 2 and Figure 3 show examples of stimuli used in the experiment. In a pilot version of this experiment we tested a simple target detection response in which participants pressed a button when a square target appeared. A third of pilot participants responded to more than 30% of the catch trials (‘false alarms’, i.e., trials in which no target appeared and the participants were required to refrain from responding). Because the high number of false alarms brings into question the reliability of the responses to true targets, we opted to use a discrimination task ^38^ in the reported experimental study in order to encourage participants to respond appropriately to all targets.

Stimuli appeared as grey figures on a white background unless otherwise indicated. Four rectangles were presented, two on each side of a central 0.2° × 0.2° fixation point. The rectangles were presented in the same orientation: either all horizontal or all vertical. Each rectangle subtended visual angles of 5.0° by 1°. Adjacent rectangles' outer edges were separated by a visual angle of 5.0°. Therefore, the angular distance between the two rectangles' outer edges in each hemifield was equal to the angular length of the longest side of the individual rectangles. The distance of the closest edge of any rectangle was 1.3° from the edge of the central fixation cross. The four rectangles and the fixation cross remained on the screen for the entire trial.

After 1000 ms a cue was presented for 100 ms in the form of a blackening of the outline at one end of one of the rectangles (see Figure 2). After a further 100 ms delay, the cue was followed by a target appearing for 200 ms in 1 of 3 locations relative to the cue. The target was a black T or L, measuring 0.4° × 0.4°, which could appear at any orientation. In the majority of trials (71.4%) the target appeared in the same position on the same rectangle as the cue (validly-cued trials, see Figure 3). In 28.6% of trials the target appeared at a position other than that indicated by the cue (i.e., invalid cueing), requiring a shift of attention. In these trials, the target appeared either at the opposite end of the same rectangle as the cue (within-object shift) or at the same end of the rectangle adjacent to that which the cue had appeared in (between-object shift). These shifts were confined within the visual field such that the target always appeared on the same side of the fixation cross as the cue. Within-object and between-object shifts occurred equiprobably, and the angular distance of the attentional shift was equal for both of these conditions. Distractors in the form of 0.4° × 0.4° black T/L hybrids (Fs) appeared in all other locations for the same duration as the target (200 ms).

The participant was asked to indicate whether the target was a T or an L. Responses were given by pressing either of two keys of a standard keyboard with the index and middle fingers of the right hand, with response mapping counterbalanced between participants. The response keys were placed directly in front of the participant’s body midline. The trial ended when the participant gave their response, or after 3000 ms, whichever came first. There was a 500 ms inter-trial interval in which only the fixation cross remained on the screen. Each participant completed twelve experimental blocks of 112 trials each.

The participant was instructed to respond as quickly and as accurately as possible, and to maintain central fixation. Fixation was monitored by the experimenter through a closed-circuit television. For trials in which eye movements occurred the experimenter clicked a mouse button, and these trials were later removed from analysis.

### Data analysis

The data from the open-loop pointing and Egly tasks were analysed using repeated-measures ANOVAs. Sphericity was violated in some datasets ( *p* for Mauchley’s W > .05), in which case Greenhouse-Geisser-corrected alpha levels were used and are indicated. Follow-up analyses were performed using paired- and independent-samples t-tests with Bonferroni-corrected alpha levels.

Pointing errors were analysed using a Group (L, N, R) × Session (pre, post, late) ANOVA.

For the Egly task, trials in which participants made an eye movement or gave an incorrect response were excluded from the RT analyses. In addition, because there were a substantially greater number of valid than invalid trials, each participant’s data were screened for outliers in the valid, within-object and between-object conditions, with RTs more than 2SD from the mean for each condition excluded from analysis. These criteria resulted in excluding less than 1% of the data. For each participant, mean RTs and percentage accuracy were calculated for each condition and grouped for further analysis. Three sets of repeated-measures ANOVAs were performed to examine the effects of prism adaptation on different aspects of space- and object-based attention.

First, to examine the effects of prism adaptation on object-based attention in each visual field, we performed repeated-measures ANOVAs of mean RTs and percentage accuracy with three factors: Group (L, N, R) × Visual Field [left visual field (LVF), right visual field (RVF)] × Validity (valid, within, between).

Second, to examine the effects of prism adaptation on the tonic distribution of attention across the visual field, the mean RTs for validly-cued trials were pooled according to which of the four horizontal locations they appeared in. The mean RTs and percentage accuracy were subject to Group (L, N, R) × Horizontal Location (left-LVF, right-LVF, left-RVF, right-RVF) ANOVAs with the prediction that adaptation to leftward-shifting prisms would result in slower RTs and lower accuracy in the LVF, and that this decrement in performance would be most evident in the leftmost location in the LVF.

The third set of analyses tested for any effects of prism adaptation on location-based costs of shifting attention horizontally within an object or between two objects in each visual field. The data for invalidly-cued trials involving a horizontal shift in attention were subjected to two Group (L, N, R) × Visual Field (left, right) × Shift Type (between, within) × Horizontal Shift Direction (left shift, right shift) ANOVAs. If adaptation to leftward-shifting prisms results in a neglect-like disengage deficit, participants in the leftward-shifting group would demonstrate increased RT costs and/or reduced accuracy for shifting attention leftward, which could be observed only for between-object shifts.

## Results

### Data screening

Three participants performed no better than chance in the discrimination task for the invalidly-cued trials in the Egly task (chi-squares *p*>0.05), and were excluded from further analyses.

### Pointing errors

**Figure 4.**
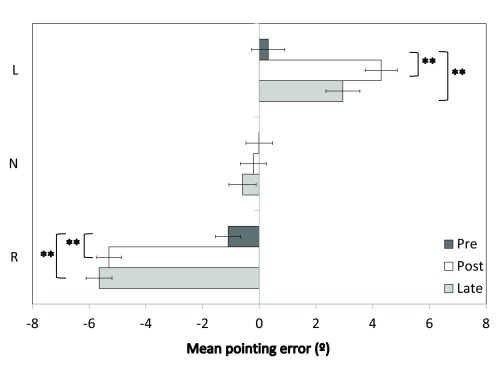
Open loop pointing errors. Horizontal pointing errors (°) are shown for the Group × Session interaction. L = leftward-shifting; N = neutral pointing; R = rightward-shifting. Error bars indicate ±1SEM; ** indicates *p*<0.001.

The raw pointing error data are provided in Dataset 1. The Group (L, N, R) × Session (pre, post, late) ANOVA revealed a main effect of Session [ *F*(1.7,93.7)=7.4, *p*<0.005, Greenhouse-Geisser corrected] and a main effect of Group [ *F*(1,54)=53.2, *p*<0.001]. These were driven by a significant Group × Session interaction [ *F*(3.4,93.7)=61.0, *p*<0.001, Greenhouse-Geisser corrected], which is plotted in Figure 4.

Dataset 1. Mean pointing errors Pointing errors (º) were measured for each individual participant in sets of 12 trials before (pre-test) and after (post-test) the first set of prism adaptation, and at the end of the experimental session (late-test). For each participant the errors were averaged within each pointing block and are provided here. L = leftward-shifting prism group; R = rightward-shifting prism group; N=neutral pointing group.

The mean pointing errors for the neutral group were unchanged across sessions (Pre: *M*=0.0, *SEM*=0.43; Post: *M*=-0.2, *SEM*=0.46; Late: *M*=-0.6, *SEM*=0.57; *p*s>0.05). For the leftward-shifting prism group there was a significant rightward shift in pointing errors between the pre-adaptation ( *M*=0.4, *SEM*=0.44) and post-adaptation sessions [ *M*=4.3, *SEM*=0.47; *t*(18)=9.6, *p*<0.001]. Pointing errors at the late test ( *M*=3.0, *SEM*=0.58) were also significantly rightward of the pre-adaptation error level [ *t*(18)=4.7, *p*<0.001].

Similarly, for the rightward-shifting prism group there was a significant leftward shift in pointing errors between pre-adaptation ( *M*=-1.1, *SEM*=0.46) and post-adaptation [ *M*=-5.3, *SEM*=0.48; *t*(19)=13.5, *p*<0.001]. Pointing errors at the late test were also significantly leftward of pre-test error [ *M*=-5.7, *SEM*=0.60; *t*(19)=14.2, *p*<0.001].

In summary, as expected, there was no change in pointing error for the neutral group, and the leftward- and rightward-shifting prism groups showed significant changes in pointing errors that were in the predicted direction for the prismatic shift and were sustained throughout the entire post-adaptation Egly task.

### Egly task

The analyses of accuracy and mean RTs produced identical results with respect to the experimental hypotheses. For the sake of brevity, only the RT analyses are reported here, however both raw datasets are provided ( Dataset 2– Dataset 7). Mean accuracy was 85%.

**Figure 5.**
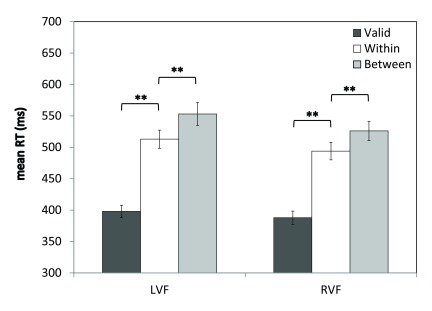
Visual field × Validity interaction. Mean RTs (ms) are shown for the three different target types in the left visual field (LVF) and right visual field (RVF). Error bars indicate ±1SEM; ** indicates *p*<0.001.

The Group (N, L, R) × Visual Field (left, right) × Validity (valid, within, between) ANOVA revealed a main effect of Visual Field [ *F*(1,54)=20.0, *p*<0.001] and a main effect of Validity [ *F*(2,108)=199.0, *p*<0.001]. These were driven by a significant Visual Field × Validity interaction [ *F*(2,104.5)=3.36, *p*<0.05; Greenhouse-Geisser corrected], which is shown in Figure 5. In the LVF, RTs for validly-cued trials were 115 ms faster than RTs for trials requiring a within-object shift of attention [valid: *M*=398, *SEM*=10; within-object: *M*=513, *SEM*=15; *t*(56)=15.0, *p*<0.001], and trials requiring a between-object shift in attention ( *M*=553, *SEM*=18) were a further 40 ms slower compared to within-object shifts [ *t*(56)=6.2, *p*<0.001]. In the RVF, RTs for validly-cued trials were 106 ms faster than for the within-object shift condition [valid: *M*=388, *SEM*=10.6; within-object: *M*=494, *SEM*=14; *t*(56)=14.5, *p*<0.001], and RTs for the between-object shift condition ( *M*=526, *SEM*=16) were a further 32 ms slower than for trials requiring a within-object shift of attention [ *t*(56)=5.3, *p*<0.001]. Finally, for each of the three trial types, RTs were significantly faster for trials in the RVF compared to the LVF ( *p*s<0.005). There were no further significant main effects or interactions, including no main effects or interactions involving Group ( *F*s<2.1, *p*s>0.05). The raw percentage accuracy and mean RTs for this analysis are provided in Datasets 2 and 3.

Datasets 2 and 3: Percentage accuracy and mean RTs (ms) for the group by visual field by validity ANOVA. Dataset 2: For each participant, percentage accuracy was calculated within each of the levels of visual field and validity. The group data were subjected to a repeated measures ANOVA. The analyses of percentage accuracy are not reported, as they resulted in similar results with regards to the experimental hypotheses as the analysis of mean RTs. L = leftward-shifting prism group; R = rightward-shifting prism group; N=neutral pointing group; LVF = left visual field; RVF = right visual field.
Dataset 3: For each participant, RTs (ms) were averaged within each of the levels of visual field and validity. The group data were subjected to a repeated measures ANOVA, as reported in the text. L = leftward-shifting prism group; R = rightward-shifting prism group; N=neutral pointing group; LVF = left visual field; RVF = right visual field.

These results replicate the classic Egly effect: invalidly-cued trials are associated with slower RTs compared to validly-cued trials, and this RT cost is significantly larger for between-object shifts of attention than within-object shifts. This pattern was present in both visual fields and was unaltered by prism adaptation.

**Figure 6.**
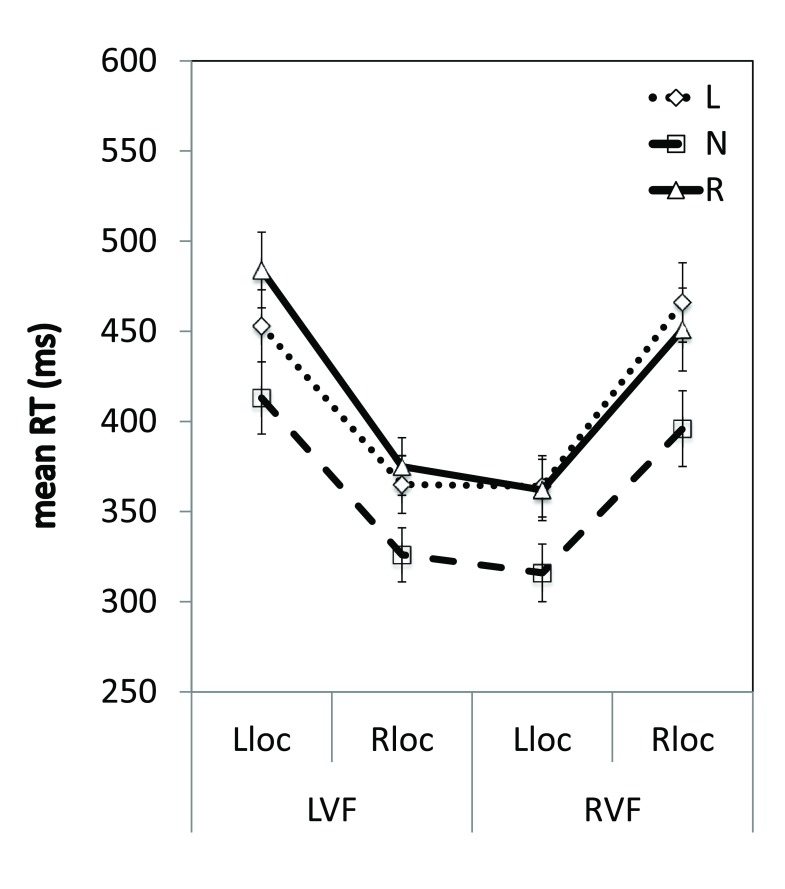
Reaction times across the visual field. Mean RTs (ms) are shown for validly-cued trials according to horizontal location for the three different groups. L = leftward-shifting; R = rightward-shifting, N = neutral pointing; LVF = left visual field; RVF = right visual field; Lloc = left location, Rloc = right location. Error bars indicate ±1SEM.

The percentage accuracy and mean RT data of each participant for the Group (L, N, R) × Horizontal Location (left-LVF, right-LVF, left-RVF, right-RVF) analyses are provided in Datasets 4 and 5. The ANOVA of mean RTs revealed a significant main effect of Horizontal Location [ *F*(1,105)=116.0, *p*<0.001], and a non-significant trend for the Group × Horizontal Location interaction [ *F*(3.1,84.7)=2.6, *p*=0.054; Greenhouse-Geisser corrected]. Paired-samples t-tests revealed that in each group the RTs for targets appearing closer to fixation (i.e., right-LVF and left-RVF) were significantly faster than those appearing further away to fixation (i.e., left-LVF and right-RVF, *p*s<0.001; Figure 6).

Datasets 4 and 5: Percentage accuracy and mean RTs (ms) for the group by horizontal location ANOVA. Dataset 4: For each participant, percentage accuracy was calculated within each of the four horizontal location conditions. The group data were subjected to a repeated measures ANOVA. The analyses of percentage accuracy are not reported, as they resulted in similar results with regards to the experimental hypotheses as the analysis of mean RTs. L = leftward-shifting prism group; R = rightward-shifting prism group; N=neutral pointing group; LVF = left visual field; RVF = right visual field; leftloc = left location; rightloc = right location.
Dataset 5: For each participant, RTs (ms) were averaged within each of the four horizontal location conditions. The group data were subjected to a repeated measures ANOVA, as reported in the text. L = leftward-shifting prism group; R = rightward-shifting prism group; N=neutral pointing group; LVF = left visual field; RVF = right visual field; leftloc = left location; rightloc = right location.

Independent-samples t-tests comparing the mean RTs for the three groups at each location revealed that the leftward-shifting prism group responded to targets in the rightmost location of the RVF 70 ms slower than the neutral pointing group [left: *M*=466, *SEM*=80.4; neutral: *M*=396, *SEM*=79.8; *t*(37)=2.7, *p*<0.012], however there were no significant differences in the RTs of these groups in the three other locations ( *p*s>0.012, Bonferroni corrected). There were also no significant differences between the leftward- and rightward-shifting prism groups, or between the rightward-shifting prism group and the neutral pointing group in any of the four locations ( *p*s>0.012, Bonferroni corrected). Therefore, these results are not consistent with a systematic neglect-like change in the RTs of the leftward-shifting prism group, but instead reflect a typical eccentricity-driven distribution of attention ^39– 41^.

**Figure 7.**
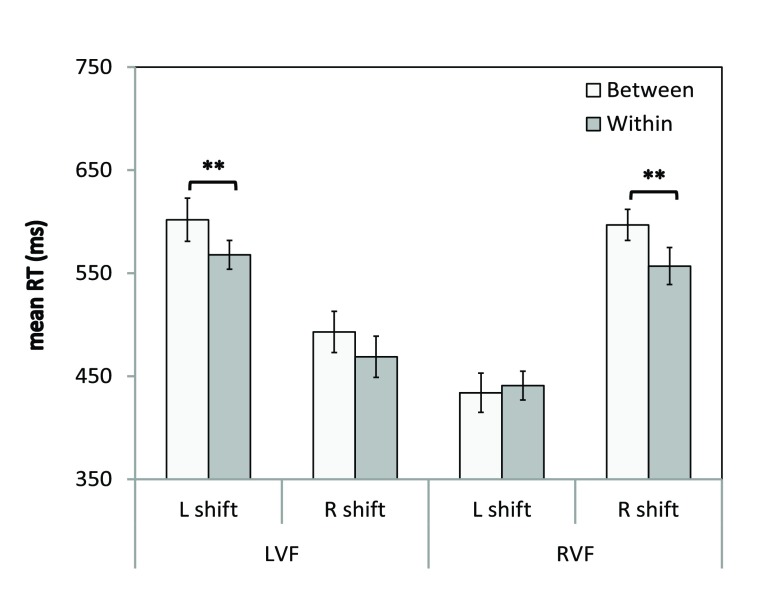
Visual field × Shift Type × Horizontal Shift Direction interaction. Mean RTs (ms) are shown for invalidly-cued trials that involved a horizontal shift in attention. LVF = left visual field; RVF = right visual field; L shift = left shift; R shift = right shift. Error bars indicate ±1SEM; ** indicates *p*<0.005 for each t-test comparison of between- and within-object shifts.

The Group (N, L, R) × Visual Field (LVF, RVF) × Shift Type (between, within) × Horizontal Shift Direction (left-shift, right-shift) ANOVA was performed on the mean RTs for invalidly-cued trials only. There was a significant main effect of Visual Field [ *F*(1,54)=9.0, *p*<0.005], a significant main effect of Shift Type [ *F*(1,54)=28.2, *p*<0.002] and a significant main effect of Horizontal Shift Direction [ *F*(1,54)=4.7, *p*<0.05]. There was also a significant Visual Field × Horizontal Shift Direction interaction [ *F*(1,54)=170.7, *p*<0.005]. These were driven by a significant three-way interaction of Visual Field × Shift Type × Horizontal Shift Direction [ *F*(1, 54)=4.3, *p*<0.05), which is plotted in Figure 7. The percentage accuracy and mean RTs for each participant for this analysis is provided in Datasets 6 and 7.

Datasets 6 and 7: Percentage accuracy and mean RTs (ms) for the group by visual field x shift type x horizontal shift direction ANOVA. Dataset 6: For each participant, percentage accuracy was calculated within each level of visual field and horizontal shift direction. The group data were subjected to a repeated measures ANOVA. The analyses of percentage accuracy are not reported, as they resulted in similar results with regards to the experimental hypotheses as the analysis of mean RTs. L = leftward-shifting prism group; R = rightward-shifting prism group; N=neutral pointing group; LVF = left visual field; RVF = right visual field.
Dataset 7: For each participant, RTs (ms) were averaged within each level of visual field and horizontal shift direction. The group data were subjected to a repeated measures ANOVA, as reported in the text. L = leftward-shifting prism group; R = rightward-shifting prism group; N=neutral pointing group; LVF = left visual field; RVF = right visual field;

For between-object shifts, mean RTs for shifting rightward in the LVF were 109 ms faster than mean RTs for shifting leftward in the LVF [leftward: *M*=602, *SEM*=21.4; rightward: *M*=493, *SEM*=20; *t*(56)=8.6, *p*<0.001]. Similarly, for between-object shifts, mean RTs for shifting leftward in the RVF were 143 ms faster than mean RTs for shifting rightward in the RVF [leftward: *M*=454, *SEM*=14; rightward: *M*=597, *SEM*=19.8; *t*(56)=10.9, *p*<0.001]. For within-object shifts, mean RTs for shifting rightward in the LVF were 99 ms faster than mean RTs for shifting leftward in the LVF [leftward: *M*=568, *SEM*=18.5; rightward: *M*=469, *SEM*=14.7; *t*(56)=9.2, *p*<0.001]. Similarly, for within-object shifts, mean RTs for shifting leftward in the RVF were 116 ms faster than mean RTs for shifting rightward in the RVF [leftward: *M*=440, *SEM*=14.0; rightward: *M*=556, *SEM*=18.8; *t*(56)=9.7, *p*<0.001]. Furthermore, t-tests comparing RTs for between- to within-object shifts for each shift direction in each visual field revealed that the RT advantage for within-object shifts was only significant for shifts away from fixation ( *p*s<0.005). Finally, there were no main effects or interactions involving Group ( *F*s<2.0, *p*s>0.05).

## Discussion

The results demonstrate that adaptation to leftward-shifting prisms did not alter the performance of healthy participants on the Egly task. Specifically, there were no differences between the three groups in their relative RTs for validly-cued, within-object and between-object trials ( Figure 5), indicating no effect of prism adaptation on object-based attention. RTs for validly-cued trials in four locations across the visual field were numerically larger for both prism groups compared to the neutral pointing group ( Figure 6). However, this difference was not significant, and the responses of the leftward-shifting prism group were not consistent with a neglect-like decrement in LVF attention relative to the neutral group. Finally, there were no differences between the three groups in the speed with which attention was shifted laterally in each visual field ( Figure 7), indicating that adaptation to leftward-shifting prisms did not produce a neglect-like disengage deficit. Therefore, although significant motor after-effects were obtained for both prism groups, the data do not provide any evidence that prism adaptation alters space- or object-based attention in healthy participants.

The existing literature provides a number of examples of tasks for which prism adaptation has been shown to improve the performance of neglect patients and also produce neglect-like changes in the performance of healthy participants: for example line bisection ^13, 19, 42^, mental number bisection ^14, 43^, haptic circle centring ^5, 17^ and postural control ^10, 18^. However, there are some tasks for which prism adaptation has been shown to reduce neglect symptoms, but has no impact on the performance of healthy participants: visual search ^27, 44^ and a temporal order judgement task sensitive to biases in spatial attention ^26^. Our results are consistent with the latter category in that there is substantial evidence that prism adaptation can influence the spatial distribution of attention in neglect patients ^26, 28, 34^, and specific evidence that it influences the performance of neglect patients on the Egly task ^30^, however we find no evidence for any prism-related differences in the performance of healthy participants on our Egly task. These results are consistent with those of Schindler and colleagues ^30^, who found that adaptation to rightward-shifting prisms did not alter the performance of healthy participants on a version of the Egly task, and we have extended their findings by showing that there are also no changes following adaptation to leftward-shifting prisms.

We have previously noted that any aspects of performance that have been altered by prism adaptation in healthy individuals are ones for which ‘normal’ behaviour is already biased ^45^. The term ‘pseudoneglect’ refers to the commonly observed leftward bias that is shown by healthy participants on visuo-spatial tasks such as line bisection, even in the absence of motor responses ^46^. One explanation for this bias is that engaging in visuo-spatial tasks, which favour right-hemisphere processing, results in a slight imbalance in the opponent attentional gradients of the two hemispheres such that the leftward orienting controlled by the right hemisphere is dominant. Many studies that demonstrate changes in the performance of healthy participants following prism adaptation could be interpreted as ‘treating’ or reducing pseudoneglect rather than inducing neglect-like patterns ^13, 15, 16, 26^. Consistent with this explanation, we demonstrated that prism adaptation altered the extent to which participants processed the local elements of a visual image compared to the global configuration ^45^. Like pseudoneglect, global-local processing has been related to opponent processes of the left and right hemispheres ^47, 48^, and healthy participants normally show a bias in favour of right-hemisphere global processing ^49^.

In contrast, healthy participants do not normally show a pseudoneglect-style spatial bias when performing the Egly task , visual search ^44^ or temporal order judgement ^50^. Although the participants in the present study did show an overall spatial bias (see Figure 4), this was present as a RVF RT advantage, rather than a LVF RT advantage as would be indicative of pseudoneglect. Because all participants gave their responses using their right hands, the RVF advantage can be attributed to the common phenomenon of speeded responses when stimuli are presented in the same side of space as the response button or responding hand (the ‘Simon effect’ ^51^). That is, the RT imbalance that we observed is probably not due to a hemispheric or visual field difference in the way that the central cognitive task is performed per se. Our results are therefore consistent with our previous proposal that prism adaptation is more likely to perturb cognitive functions for which the baseline performance is already biased (either by brain damage or due to normal cognitive phenomena such as pseudoneglect ^45^).

Assuming that the mechanisms through which prism adaptation influences the performance of healthy participants and neglect patients are similar, it is possible that the absence of any effects of left-shifting prisms in inducing a neglect-like effect on healthy participants in the current study can be attributed to our design. Schindler and colleagues ^30^ used a simple detection task to demonstrate the benefits of rightward-shifting prisms for patients with neglect whereas the present study used a discrimination task. It is possible that prism adaptation influences only early or rapid attentional processes and changes in performance do not manifest in the longer time period that it takes to identify a target. Furthermore, we restricted the cues and targets for each trial within a visual field. Prism adaptation may only influence object-based attention in situations in which attention is shifted between objects in different visual fields (e.g., due to the demand for interhemispheric communication). In the present study, limiting the stimuli for each trial to within a visual hemifield may have meant that once cued, attention remained focussed within the visual field so that the demands of shifting attention to a different object in the same visual field were smaller than what would be required for a between-object shift *across* visual fields. A smaller overall effect of object-based attention would mean that any differences between the groups could be too small to detect.

Prism effects aside, our study extended the findings of Egly and colleagues ^23^ by demonstrating that the RT cost associated with shifting attention between-objects compared to within-objects are present when each visual field is probed separately. Furthermore, for horizontal shifts of attention these differences are only significant when shifting attention outward, away from fixation. This adds to an extensive literature supporting the existence of object-based attention ^21, 22, 52^.

To understand the cognitive and neural mechanisms that underlie prism adaptation, it is important to examine tasks on which this technique has no impact, as well as those for which changes are observed. Our results show that prism adaptation does not alter object- or space-based attention in healthy participants performing the Egly task. Although it is possible that particular features of our design, such as the use of a discrimination task rather than a detection task, contributed to this null finding, our results are also consistent with the proposal that prism adaptation may only perturb cognitive functions for which normal baseline performance is already biased.
