# Supplementary material for: Prism adaptation does not alter object-based attention in healthy participants
Source: F1000Res. 2013 Nov 4;2:232. [Version 1] doi: 10.12688/f1000research.2-232.v1 (PMC3962007; doi:10.12688/f1000research.2-232.v1)
Supplement: Peer review history

# Peer review: Referee response for version 1

**Published:** 2014-03-07

**Authors:** Igor Schindler

**Affiliations:** 1 Department of Psychology, University of Hull, Hull, UK

**General**

This is a well designed study using appropriate methods. The authors demonstrate convincingly that prism adaptation does not impact on either within or between object attention shifts. The introduction is, overall, scholarly and concise and the discussion provides a comprehensive explanation of the findings by arguing that a biased normal baseline performance is a pre-requisite for observing significant changes in attention biases following prism adaptation. I only have a few very minor suggestions/comments the authors may want to consider:

**Introduction**

As Radek Ptak pointed out not all studies using prism adaptation in neglect yielded positive results.

I agree regarding the inclusion of conflicting results but it is clear that the methods used varied between studies such as the extent to which the hand/arm was seen by the subject during pointing (full view of the arm vs. view of the finger only during adaptation). In addition, the type of prisms used varied across studies i.e. some used Fresnel prisms that do not provide a seamless shift of the visual field whereas others used prisms that fused magnifying lenses with prism lenses providing a seamless visual percept.

Finally, the nature of brain damage itself may account for some patients showing larger prismatic after-effects than others. A recent fMRI study by Luauté et al. (2009) in healthy participants indicated increased activation of inferior parietal areas during the early stage of error detection and correction and temporal areas during the later stage of sensory realignment. Thus the combination of different prisms, adaptation protocols and lesion location/extent as well as lesion age might contribute in part to these conflicting findings.

**Results**

On page 7 it is stated that the mean accuracy was 85% which could be further specified by including the accuracy range across adaptation groups to provide information on whether the accuracy levels were comparable.

Also one interesting finding is that the neutral group seems to show faster reaction times compared to both prism groups irrespective of target location in the visual field. It would be useful to evaluate whether the two prism groups (pooled) showed overall higher reaction times than the neutral group as suggested by Radek Ptak ****but also whether the increase in reaction times was paralleled by a change in accuracy. If this is the case, it would suggest a perturbing overall impact of prism adaptation in healthy participants in the Egly task. This would potentially strengthen the argument of non–beneficial effects of prism adaptation in tasks that elicit unbiased performance in healthy participants.

I have read this submission. I believe that I have an appropriate level of expertise to confirm that it is of an acceptable scientific standard.

# Peer review: Referee response for version 1

**Published:** 2014-01-17

**Authors:** Radek Ptak

**Affiliations:** 1 Division of Neurohabilitation, Geneva University Hospital, Geneva, Switzerland

This well-written paper presents a methodologically sound study that examined the effects of prism adaptation on within- and between-object shifts of attention in healthy participants. The main finding is negative, namely that prism adaptation neither affects space-based nor object-based attention. This finding was observed despite robust adaptation effects found in open-loop pointing. The conclusion of the authors is that prism adaptation may ‘only perturb cognitive functions for which normal baseline performance is already biased’, which according to them is not the case for the task used in the present study.

 

The title of the article is appropriate and the abstract adequately summarizes the main findings. I recommend this article, but have three minor comments concerning the introduction, methods and interpretation of the results.

 

**Introduction**

The authors should be cautious when discussing the beneficial effects of prism adaptation on spatial neglect. Rehabilitation of neglect certainly is a great challenge and therapeutic techniques that appear to have lasting effects following very short interventions should undergo rigorous assessment with randomized, controlled clinical trials. It should be noted that the positive results of Rossetti *et al.* (1998), were not replicated in a more recent study using the same methodology ( Rousseaux *et al.*, 2006). In addition, a randomized study on a much larger group of neglect patients did not find any benefits of prism adaptation, as compared to repeated pointing with sham prisms ( Turton *et al.*, 2010).

I think that in the domain of rehabilitation negative results are at least as important as positive results, and should therefore be acknowledged by the authors.

 

**Methods**

The fact that all participants responded with their right hand in the RT task is problematic, as small effects due to prism adaptation might be masked by larger effects due to space-hand mapping. I appreciate that the authors are aware of this limitation.

 

**Results**

I was surprised that the analysis of results presented in Figure 6 did not yield a main group effect (the authors only describe a marginal group x horizontal location interaction). Interestingly, both adaptation groups show increased RTs when compared to the non-adapted group, an effect that is consistent across all four locations. What was the p-value of the main group effect in the ANOVA and is there a possibility to further examine this group effect (e.g., by pooling the two prism adaptation groups and comparing them to the non-adapted group)? I think this slowing of RTs following prism adaptation, irrespective of direction of the adaptation, is worth at least a comment if not further study.

 

***Suggested literature***

Rousseaux M., Bernati T., Saj A., Kozlowski O., 2006. Ineffectiveness of prism adaptation on spatial neglect signs. *Stroke* **37**, 542-543.

Turton A.J., O'Leary K., Gabb J., Woodward R., Gilchrist I.D., 2010. A single blinded randomised controlled pilot trial of prism adaptation for improving self-care in stroke patients with neglect. *Neuropsychol Rehabil* **20**, 180-196.

I have read this submission. I believe that I have an appropriate level of expertise to confirm that it is of an acceptable scientific standard.

# Peer review: Referee response for version 1

**Published:** 2013-12-18

**Authors:** Daniel Smith

**Affiliations:** 1 Department of Psychology, Durham University, Durham, UK

This is a well written paper describing a rigorously conducted study. The title accurately reflects the results and the abstract does a good job of summarizing the key results. It's not clear from the introduction whether patients experience attenuation of neglect only when wearing the prisms, or whether the effects persist after removal of the prisms. Perhaps the authors could add a sentence or two to clarify?  My only comment on the results is that I would like to see estimates of effect sizes reported to help interpret the data. The conclusions are justified given the data.

I have read this submission. I believe that I have an appropriate level of expertise to confirm that it is of an acceptable scientific standard.

# Response

**Published:** 2013-12-21

**Authors:** Janet Bultitude

**Affiliations:** University of Oxford, UK

Many thanks for these comments and suggestions. The journal editors have suggested that we await further reviewer comments before amending the text of the article. In the mean-time I am happy to address your comments here.

Patients with hemispatient neglect demonstrate reduced symptoms after undergoing prism adaptation, and this amelioration generally persists for at least two hours after a single treatment session ( Rossetti *et al*., 1998), and in some studies has been reported to persist for as much as a day ( Farné *et al.*, 2002) or even a week ( McIntosh *et al*., 2002) following prism adaptation.

We will revise the results section to include effect sizes.
